# Ictal semiology in temporo‐frontal epilepsy: A systematic review and meta‐analysis

**DOI:** 10.1002/epd2.20328

**Published:** 2024-12-26

**Authors:** Irina Oane, Andrei Barborica, Ioana Mîndruţă

**Affiliations:** ^1^ Neurology Department, Epilepsy Monitoring Unit University Emergency Hospital Bucharest Bucharest Romania; ^2^ Physics Department University of Bucharest Bucharest Romania; ^3^ Clinical Neurosciences Department Carol Davila University of Medicine and Pharmacy Bucharest Romania

**Keywords:** anatomo‐clinical correlation, epilepsy surgery, ictal semiology, SEEG, systematic review, temporo‐frontal epilepsy

## Abstract

We performed a systematic review of the ictal semiology of temporo‐frontal seizures with the aim to summarize the state‐of‐the‐art anatomo‐clinical correlations in the field, and help guide the interpretation of ictal semiology within the framework of presurgical evaluation. We conducted the systematic review and meta‐analysis, and reported its results according to the Preferred Reporting Items for Systematic Review and Meta‐Analysis statement. We searched electronic databases (Scopus, PUBMED, Web of Science, and EMBASE) using relevant keywords related to temporal, frontal and sublobar structures, semiology, and electroencephalography/stereoelectroencephalography exploration. The risk of bias was evaluated using the QUADAS2. We included articles in English, reporting the seizure semiology of patients with temporal lobe epilepsy with temporal‐frontal involvement and patients with frontal lobe epilepsy and fronto‐temporal network involved. We performed hierarchical cluster analysis to determine signs and symptoms associated with the temporo‐frontal epileptogenic network for all patients and for each subgroup (frontal/temporal seizure onset). Fisher exact test was performed to evaluate the difference in seizure freedom and clinical sign/symptom occurrence in patients that underwent unilobar versus bilobar resection. Meta‐analysis on the prevalence of temporo‐frontal/fronto‐temporal involvement applying a random‐effect model was used. We included 40 articles and we extracted data from 109 patients. The meta‐analysis showed the total prevalence of temporo‐frontal/fronto‐temporal network involvement was 19.75%, CI 12.02–27.47, high heterogeneity (82.71%). For the whole group and subgroups, the main cluster of clinical manifestations is emotional, autonomic, cognitive, grimace, hyperkinetic (association coefficient higher than .6). Elementary motor semiology is significantly associated with multilobar resection (*p* = .022 whole group and *p* = .0012 fronto‐temporal subgroup). Fifty‐eight patients were seizure‐free after surgery. There was no significant difference between seizure freedom in uni versus bilobar resections (*p* = .28). Seizures involving temporo‐frontal/fronto‐temporal network usually manifest with a cluster of signs and symptoms: emotional, autonomic, grimace, cognitive and hyperkinetic behavior. Based on semiology, one cannot distinguish between fronto‐temporal and temporo‐frontal cases at individual patient level. In those patients undergoing a surgical procedure, elementary motor seizure semiology is significantly associated with multilobar resection.

**Key points**

- This systematic review and meta‐analysis includes 40 articles with a total number of 109 patients reported.
- Our aim was to describe semiologic clinical characteristics of seizures in patients with fronto‐temporal and temporo‐frontal epilepsy.
- Among the ictal signs that we encountered are those that we can include in the following categories: automatisms, elementary motor and hyperkinetic behavior, autonomic symptoms, emotional abnormalities, grimacing, and cognitive impairment.
- The autonomic and emotional symptoms are present at the onset of seizure, whereas automatisms, hyperkinetic behavior and grimacing are related to the propagation phase.
- Based on semiology only, at individual patient level, one cannot distinguish between fronto‐temporal and temporo‐frontal cases.
- The cluster analysis shows a first cluster containing emotional, grimacing, autonomic, cognitive, and hyperkinetic signs with high association coefficients in all subgroups.
- The main network of temporo‐frontal propagation involves anterior‐medial temporal lobe structures and fronto‐bazal/frontal‐mesial structures.
- Patients receiving multilobar resections (all group and fronto‐temporal subgroup) displayed statistically significant more frequent elementary motor signs.

## 1 INTRODUCTION

Temporal lobe epilepsy is the most common type of focal epilepsy amendable to surgical treatment with better postsurgical outcome in terms of seizure freedom compared with other lobar localizations. Before surgery, it is helpful to map the pathway involved by the seizure spread particularly toward the frontal lobe. The first studies addressing the issue of temporal‐frontal seizure propagation using invasive recordings highlight that the most common spread of ictal activity from the temporal lobe to the ipsilateral frontal then contralateral frontal and lastly the contralateral temporal particularly affecting the mesial temporal lobe and the orbitofrontal cortex.^1^ Furthermore, temporal lobe seizures, even if focal and confined to the mesial structures, impact large brain networks such as frontal–parietal. The slow activity involving these structures in the ictal and postictal phases is contributing to the loss of consciousness and behavior changes.^2^

However, for several decades, it has been shown that the proportion of patients remaining seizure‐free at >5 years post‐surgery falls at 66% because of the complex organization of the epileptogenic zone extending outside the temporal lobe, particularly to the frontal, insular, or temporo‐parieto‐occipital junction lobe.^3^ Thus, the concept of temporal plus epilepsy has been introduced. From an anatomo‐electroclinical perspective, in these patients, seizure semiology consists more often of gustatory, vestibular, or auditory symptoms, contraversive manifestations of the eyes or head, piloerection, and ipsilateral tonic motor signs, and are more dysphoric during the post‐ictal phase. The morphology of the ictal discharge that starts with fast activity and early involvement of temporal as well as extratemporal structures simultaneously, suggests an extended multilobar epileptogenicity as quantified by several computational methods.^4^ In these particular cases, invasive recordings using stereo‐EEG electrodes are desirable being the only ones suitable to explore deep‐seated structures like the mesial temporal lobe structures, insula cortex, and orbitofrontal cortex^5^ based on a network hypothesis.

We thus performed a systematic review of the ictal semiology of temporo‐frontal and fronto‐temporal seizures in focal epilepsy with the view to summarize the state‐of‐the‐art anatomo‐clinical correlations in the field and help guide interpretation of ictal semiology within the framework of presurgical evaluation.

## 2 METHODS

### 2.1 Search method and eligibility criteria

The study protocol was registered on the international prospective systematic reviews (PROSPERO; number CRD42024535612). We conducted the systematic review of the published evidence and reported its results according to the Preferred Reporting Items for Systematic Review and Meta‐Analysis (PRISMA) statement.

The aim of the review is to determine (i) the prevalence of temporo‐frontal/fronto‐temporal epilepsy in patients undergoing presurgical evaluation for temporal or frontal drug‐resistant epilepsy, (ii) specific semiology associated with a temporo‐frontal/fronto‐temporal network, (iii) specific semiology predicting a bilobar (fronto‐temporal) resection. We formulated our review question using the Participant, Intervention, Comparison, Outcome (PICO) framework as follows: “Is there a specific semiology associated with bilobar (fronto‐temporal) resections versus unilobar?”. We therefore considered: P: patients with drug‐resistant frontal or temporal lobe epilepsy, I: presurgical evaluation C: we compared semiologic features in the group of patients undergoing unilobar (only temporal or only frontal) resection versus the group of patients undergoing bilobar fronto‐temporal resection O: postsurgical seizure freedom.

We searched electronic databases (Scopus, PUBMED, Web of Science, and EMBASE) using relevant keywords related to temporal, frontal, and sublobar structures, semiology and EEG/SEEG exploration as well as MeSH suggested terms such as “epileptic disorder” or “seizure disorder” and created the following search string: tempor* AND (front* OR prefrontal OR orbitofrontal OR cingulate OR premotor) AND (surg*) AND (epilep* OR seizure* OR epilepsy disorder OR seizure disorder) AND (eeg OR electroencephalography OR seeg OR stereo‐EEG OR stereoelectroencephalography OR intracranial). The date last searched was August 21, 2024. For details see Appendix S1.

We selected studies published as articles in peer‐reviewed journals, with an abstract available, and imported them into the online Rayyan platform to allow comprehensive, transparent, and systematic reviewing. Two independent reviewers (A.B., I.O.) screened titles, abstracts, and full‐text articles for eligibility criteria. A third reviewer (I.M.) resolved disagreements at the abstract and full‐text screening phase and the data substraction phase.

### 2.2 Inclusion and exclusion criteria

We included articles in English reporting seizure semiology of patients with temporal or frontal lobe drug‐resistant epilepsy in which noninvasive or invasive presurgical work‐up identified electrophysiologic or imaging arguments for temporo‐frontal/fronto‐temporal involved. We excluded articles where there was no per‐patient description of the ictal electroencephalographic (EEG) discharge or semiology and articles that did not clearly report on semiology related to frontal/temporal propagation of a temporal/frontal lobe epilepsy.

We selected only the original articles reporting case series and case reports if they were highly demonstrative and clearly describing the ictal bidirectional temporo‐frontal involvement in connection with specific semiological features.

### 2.3 Data extraction

For each selected publications, we extracted the number of reported patients and the proportion of patients with informative data regarding the topic of this review with specific emphasis on semiology, epileptogenic network, type of surgery, and postsurgical outcome (Table S1), and all such data informing on anatomo‐clinical correlations.

We evaluated the risk of bias in each publication using a QUADAS2‐adapted assessment as follows:

#### 2.3.1 Risk of selection bias

- Was a consecutive or random sample of patients enrolled?
- Was a case–control design used?
- Did the study avoid inappropriate exclusions?
- Could the selection of patients have introduced bias?

#### 2.3.2 Risk of assessment bias

Was semiology interpreted blinded to other data?

#### 2.3.3 Reliability of the reference standard

We further assessed our level of confidence in the reported epileptogenic zone according to a recently developed method. The latter is based on the availability and findings from MRI, intracerebral EEG, and postoperative outcome, and distinguishes four levels of evidence (very high, high, moderate, and low) defined as follows:

- (1) “very high” confidence in the reported EZ for patients with Engel class IA after at least 1 year of postoperative follow‐up;
- (2) “high” confidence in the reported EZ for patients with either: (i) a well‐delineated focal lesion suspected to represent at least part of the EZ (according to the authors of the publication), or (ii) a well‐delineated EZ according to all available iEEG data (according to the authors of the publication), or (iii) an Engel class I (but not specified IA) after at least 1 year of postoperative follow‐up;
- (3) “moderate” confidence in the reported EZ for patients with MRI signs of hippocampal sclerosis or atrophy suspected to be at least part of the EZ;
- (4) “low” confidence in the reported EZ for patients whose MRI would be normal or show multilobar, multifocal, or poorly delineated lesion, or with a poorly delineated EZ according to all available iEEG data (according to the authors of the publication), or an Engel class II–IV postoperative outcome provided the entire EZ has been entirely removed. Surgical failure in patients whose suspected EZ would not have been fully removed would not be considered for grading.

If several of the above items were available and provided different levels of confidence that associated with the postoperative outcome prevailed over the iEEG and MRI findings, while iEEG conclusion would prevail over MRI findings.

For each selected articles, we indicated the proportions of patients falling into each of the above categories.

### 2.4 Overall summary of evidence

The summary of evidence was eventually assessed using the GRADE system, according to the following categories of the level of evidence:

Very low reliability: The true effect is probably markedly different from the estimated effect.

Low reliability: The true effect might be markedly different from the estimated effect.

Moderate reliability: The authors believe that the true effect is probably close to the estimated effect.

High reliability: The authors have a lot of confidence that the true effect is similar to the estimated effect.

### 2.5 Statistical analysis

Patients were divided into two subgroups. In subgroup one, temporo‐frontal, we included those patients in which the seizure onset zone was located within the temporal lobe and the epileptogenic network extended to the frontal lobe. In the second subgroup, fronto‐temporal, we included patients in whom the seizure onset zone was confined to the frontal lobe but the epileptogenic network included structures part of or whole temporal lobe.

Statistical analysis was performed on all patients and both subgroups. Fisher exact test was performed to determine if there is a significant difference in seizure freedom and clinical sign/symptom category occurrence in patients that underwent unilobar versus bilobar resection.

We performed an agglomerative hierarchical cluster analysis of the seizure semiology for all patients unrelated to surgical resection (Table S2) according to the methodology described in Groppel et al. (2000)^6^ to determine the cluster of signs and symptoms associated with fronto‐temporal, temporo‐frontal epileptogenic network. Specifically, the hierarchical clustering was performed using similarity measures between symptom's clusters the association coefficient defined as the number of matches (symptoms both present or both absent in each patient), divided by the number of patients. Processing was implemented using Matlab (MathWorks, Natick, MA) function “linkage” with “average” option that refers to using unweighted average distance (unweighted pair group method with arithmetic mean—UPGMA) for clustering.

To perform the cluster analysis, seizure semiology features were classified into eight categories: emotional (fear, anxiety, pleasure), hyperkinetic, automatisms (oroalimentary automatisms, gestural automatisms, distal or proximal automatisms, mimic automatisms), motor elementary (tonic or dystonic postures, clonia, forced version head and/or eye), grimace (pouting, smile, laughter), cognitive (aphasia, amnesia, forced thinking), and other subjective features (visual, somatosensory, olfactory, gustatory) in line with^7^ (Table S2).

Further, for articles including more than 10 patients we conducted a meta‐analysis of the prevalence of temporo‐frontal/fronto‐temporal involvement. In the primary analysis, we calculated the prevalence of temporo‐frontal/fronto‐temporal involvement in patients with temporal and frontal lobe epilepsy with its 95% confidence intervals (% CIs) applying a random‐effect model due to anticipated heterogeneity. All analyses were made with STATA 14.0 using “metaprop” command.

## 3 RESULTS

**FIGURE 1:**
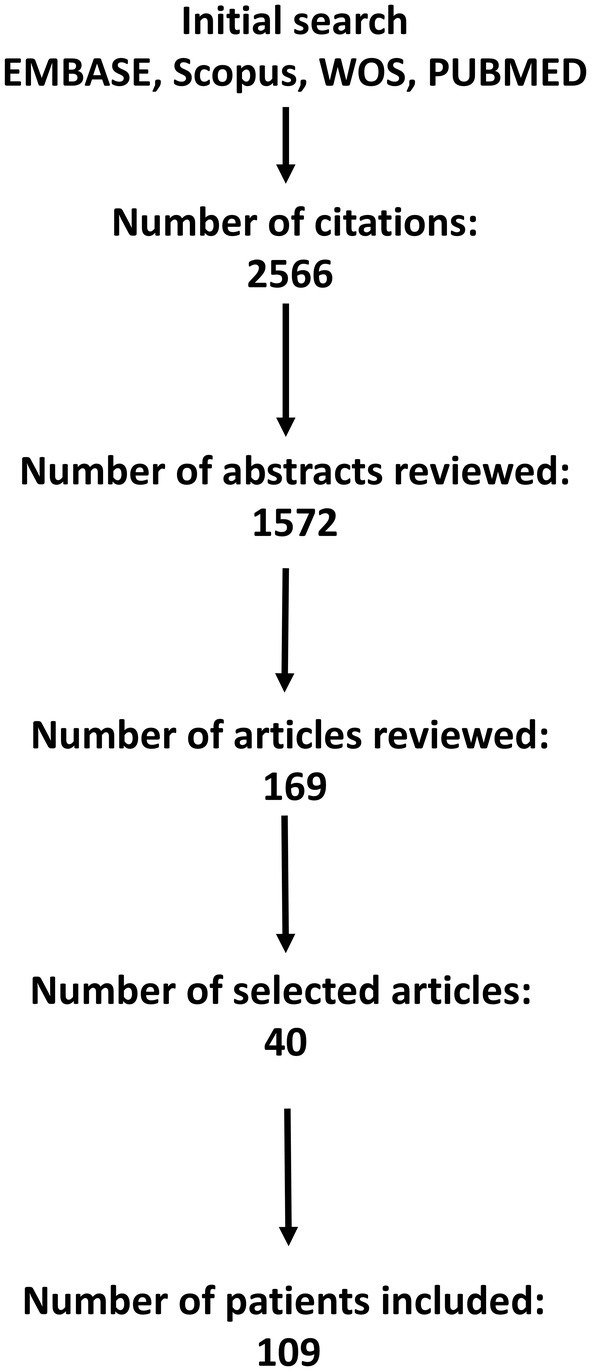
PRISMA chart.

The databases' search yielded 2566 articles and 1572 articles after resolving the duplicates. A number of 169 articles were considered potentially eligible and full‐text articles were screened to finally include 40 articles (Figure 1). Thirteen articles included more than 10 patients and 13 articles were case reports with a total number of 109 patients reported, with the median number of patients per study being 6 (1–120), the median number of patients with temporo‐frontal/fronto‐temporal involvement 1 (1–11), the median percentage of patients with lesional MRI was explored by using SEEG and operated being 100% (Table S3). There were no case–control studies, all studies reported random cases referring to a specific clinical sign such as hyperkinetic behavior or fear or to semiology in relation to a specific epilepsy localization such as temporal/frontal lobe and temporo‐perisylvian. Although there are no inappropriate case exclusions, the selection of cases could introduce bias since these articles report on specific semiology or localization. Secondly, the assessment bias is not reported in any of the publications included but we estimate it to be high since all patients included have undergone presurgical evaluation when semiology is interpreted together with imaging and electrographic results. Furthermore, the confidence in the localization of the epileptogenic zone is very high in 55% of the studies. A summary of the included studies is shown in Table 1.

**TABLE 1:** Studies included in the systematic review, number of patients per each study, number of patients displaying fronto‐temporal/temporo‐frontal epileptogenic network, MRI status, SEEG exploration, percentage of operated patients per study, type of surgery, and seizure outcome.

No.	Study	#pts	# pts. T‐F	% MRI positive	% SEEG	% operated	Type of surgery (number of patients – Type of surgery)	% class IA ≥1 year	Selection bias	Assessment bias	Reliability of EZ localization
1	Rheims et al., 2008^18^	11	4	100%	100%	100%	1 – ATL + OFC + ACC	100%	High	NR	Very high
2	Bartolomei et al., 2017^19^	1	1	0%	100%	0%	None	0%	High	NR	Low
3	Proserpio et al., 2011^20^	40	4	100%	100%	100%	2 – Temporal neocortex lesionectomy; 2 – Temporo‐basal lesionectomy	100%	High	NR	Very high
4	Nobili et al., 2002^21^	1	1	0%	100%	100%	NR	100%	High	NR	Very high
5	Biraben et al., 2001^22^	8	2	50%	100%	100%	ATL	NR	High	NR	Low
6	Bartolomei et al., 2010^23^	34	1	0%	100%	0%	None	0%	High	NR	Low
7	Vaugier et al., 2017^24^	1	1	0%	100%	0%	None	0%	High	NR	Low
8	Vaugier et al., 2009^25^	7	7	43%	100%	57%	2 – ATL, 2 – ATL + OFC, 3 – none	43%	High	NR	Moderate
9	Bartolomei et al., 2002^26^	3	3	67%	100%	0%	None	0%	High	NR	Low
10	Bottan et al., 2020^27^	6	2	50%	100%	100%	2 – ATL + OFC	100%	High	NR	Very high
11	Nobili et al., 2004^28^	3	3	100%	100%	100%	3 – ATL + extension to temporal neocortex (lesionectomy)	100%	High	NR	Very high
12	Fong et al., 2019^29^	4	1	100%	100%	100%	1 – lesionectomy (encephalocele)	100%	High	NR	Very high
13	Kuba et al., 2013^8^	120	5	80%	100%	100%	5 – ATL	100%	High	NR	Very high
14	Massot‐Tarrus et al., 2016^9^	9	4	0%	50%	50%	1 – ATL, 1 – ATL + OFC, 2 – NR	0%	High	NR	Low
15	Tassinari et al., 2005^30^	11	8	100%	0%	0%	8 – NR	0%	High	NR	Low
16	Hogan et al., 2006^31^	1	1	0%	0%	100%	1 – ATL	100%	High	NR	Very high
17	Bartolomei et al., 2002^26^	1	1	100%	100%	100%	1 – ATL	0%	High	NR	Low
18	Wang et al., 2018^32^	14	1	0%	100%	100%	1 – ATL	100%	High	NR	Very high
19	Xu et al., 2020^33^	5	2	0%	100%	100%	2 – ATL	100%	High	NR	Very high
20	Wong et al., 2010^34^	17	3	67%	0%	100%	1 – temporal neocortical lesionectomy 2 – ATL	100%	High	NR	Very high
21	Tran et al., 2014^35^	16	1	100%	0%	100%	1 – SAH	100%	High	NR	Very high
22	Loução de Amorim et al., 2020^36^	35	1	100%	0%	0%	NR	0%	High	NR	Low
23	Ozlem Dede et al., 2018^37^	15	7	100%	0%	29%	2 – SAH, 3 – ATL, 2 – NR	29%	High	NR	Moderate
24	Agashe et al., 2023^38^	1	1	100%	100%	100%	1 – temporal encephalocele	100%	High	NR	Very high
25	Liu et al., 2020^39^	4	2	0%	100%	100%	1 – OFC cortectomy 1 – RFTC OFC	100%	High	NR	Very high
26	Zhao et al., 2020^40^	27	11	64%	100%	100%	7 – OFC 2 – OFC + Amygdala 2 – OFC + insula 1 – OFC + amygdala + hippocampus	55%	High	NR	Moderate
27	Serletis et al., 2014^41^	11	6	67%	100%	100%	3 – OFC + temporal pole 2 – OFC + temporal pole + insula 1 – OFC + frontal pole + temporal posterior	100%	High	NR	Very high
28	Hedaya and Ver Hoef, 2024^42^	1	1	100%	100%	0%	NR	0%	High	NR	Low
29	Ishizaki et al., 2019^43^	1	1	100%	100%	100%	SAH	100%	High	NR	Very high
30	Sevy et al., 2014^44^	6	1	100%	100%	100%	1 – OFC + ATL	100%	High	NR	Very high
31	Alsemari et al., 2013^45^	1	1	100%	0%	100%	1 – ATL + IFG	100%	High	NR	Very high
32	Zhang et al., 2019^46^	1	1	100%	100%	100%	1 – OFC	100%	High	NR	Very high
33	Loddenkemper et al., 2003^47^	6	1	100%	0%	100%	1 – fronto‐temporal	100%	High	NR	Very high
34	Chibane et al., 2017^48^	16	4	25%	100%	100%	3 – OFC 1 – OFC + insula	100%	High	NR	Very high
35	Gurgenashvili et al., 2011^49^	1	1	100%	0%	100%	1 – temporal	0%	High	NR	Low
36	Shihabuddin et al., 2001^50^	1	1	100%	0%	100%	1 – fronto‐bazal	0%	High	NR	Low
37	Roper and Gilmore, 1995^51^	3	1	100%	0%	100%	1 – OFC + ATL	0%	High	NR	Low
38	Bottan et al.,2020^27^	6	2	50%	100%	100%	2 – OFC + ATL	10%	High	NR	Low
39	Tezer et al., 2013^52^	1	1	100%	100%	100%	1 – fronto‐bazal	100%	High	NR	Very high
40	Yu et al., 2013^53^	9	7	43%	100%	100%	7 – temporal	86%	High	NR	High

Abbreviations: ACC, anterior cingulate cortex; ATL, anterior temporal lobe; IFG, inferior frontal gyrus; NR, not reported; OFC, orbitofrontal cortex; pts., patients; RFTC, radiofrequency thermocoagulation; SAH, selective amygdalo‐hippocampectomy; SEEG, stereoelectroencephalography; T‐F, temporo‐frontal.

Based on the summary of evidence assessed by the GRADE system we evaluate the overall evidence as moderate reliability.

### 3.1 Seizure semiology

#### 3.1.1 All group

**FIGURE 2:**
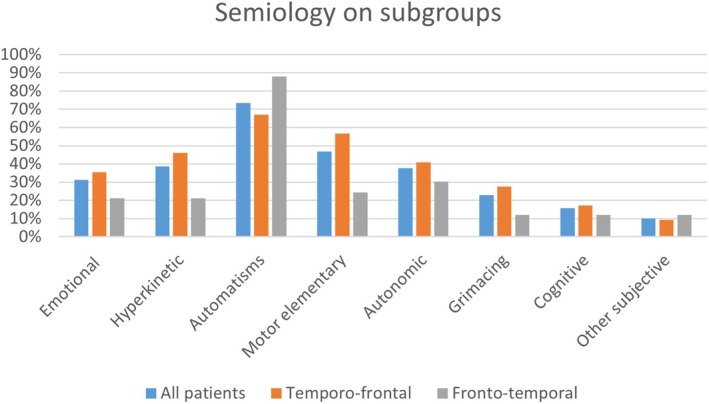
Prevalence of ictal clinical signs and symptoms for all patients group and for each subgroup (fronto‐temporal and temporo‐frontal).

**FIGURE 3:**
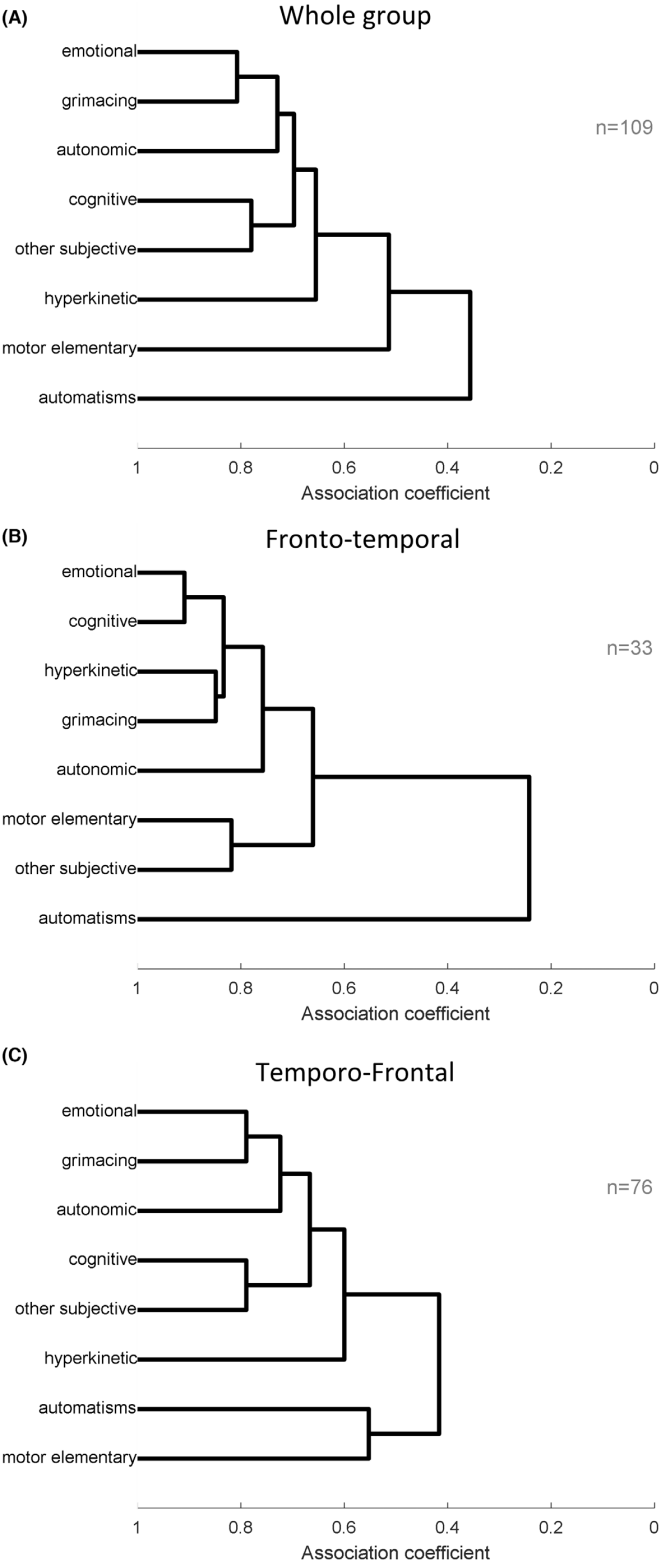
Cluster analysis for all patients group and for each subgroup, fronto‐temporal and temporo‐frontal showing that emotional, grimacing, hyperkinetic, autonomic, and cognitive clinical manifestations are included in the main cluster of symptoms for all groups.

The most frequent ictal signs were the automatisms seen in 80 patients (73%), followed by the elementary motor (47%), hyperkinetic (39%), autonomic (38%), emotional (31%), grimace (23%), cognitive (16%), and other subjective features (10%) (Figure 2). The emotional and the facial signs (grimace) have the highest association coefficient .807 (Table S4). They are followed by cognitive features and other subjective symptoms such as gustatory/olfactory with an association coefficient of .780 (Table S4) and Figure 3A. The next ones closely associated are the autonomic and hyperkinetic features with an association coefficient of .606 (Figure 3A). A second cluster is represented by the motor elementary sign with an association coefficient of around .5 and the last cluster of an isolated symptom is the automatisms with an associated coefficient higher than .3 (Figure 3A).

#### 3.1.2 Fronto‐temporal subgroup

This subgroup included 33 patients and most of them presented automatisms (88%), autonomic (30%), motor elementary signs (24%), emotional and hyperkinetic (21%), and grimacing and cognitive (12%) (Figure 2). Cluster analysis identifies the emotional and cognitive symptoms most frequently associated with a coefficient of .909 (Table S4) followed by emotional‐hyperkinetic (.879), emotional‐grimacing (.848), and emotional‐autonomic (0.0.788) (Figure 3B and Table S4). Finally, the motor elementary and other subjective symptoms appear most frequently together as ictal semiology in a subgroup of patients with an association coefficient of .818. The automatisms represent a third cluster with an isolated symptom and an association coefficient higher than .2 (Figure 3B).

#### 3.1.3 Temporo‐frontal subgroup

In this category (76 patients), the most frequently encountered ictal signs and symptoms were the automatisms (67%), motor elementary (57%), hyperkinetic (46%), emotional (36%), autonomic (41%), grimacing (28%), and cognitive (17%) similar with the results on all group patients (Figure 2). The cluster analysis follows the same organization as the whole group analysis with emotional and grimaces most frequently associated followed by autonomic, cognitive and other subjective features with a coefficient higher than .6 (Figure 3C). However, in this group, there is a second cluster of signs, the automatisms and motor elementary signs (association coefficient .553) (Figure 3C and Table S4).

For all groups, the autonomic and emotional features are present at the onset of seizure whereas automatisms, hyperkinetic behavior, and grimace are present at propagation.

In the group of patients that received multilobar surgical resection, there is a significant statistic higher occurrence of motor elementary signs if we consider all patients (*p* = .022) and also in the fronto‐temporal subgroup (*p* = .12). No other semiological category reached the statistical significance in all patients or in other subgroups.

### 3.2 Network analysis

**FIGURE 4:**
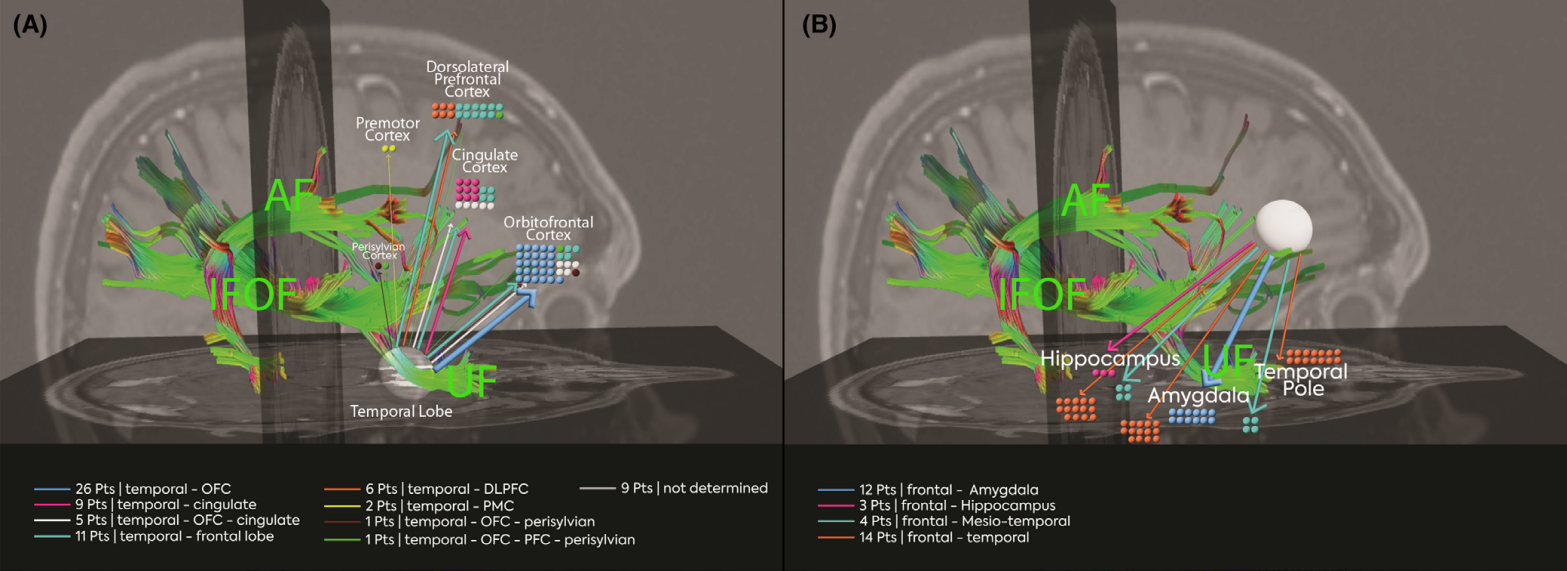
Fronto‐temporal network display. Tracts generated in DSI Studio application using specific ROI to identify main temporo‐frontal structural connections relevant for seizure propagation according to SEEG data. (A) Patients with temporal lobe epilepsy and the frontal sublobar propagation. (B) Patients with frontal lobe epilepsy and temporal sublobar propagation. AF, arcuate fasciculus; IFOF, inferior fronto‐occipital fasciculus.

70% of the patients were diagnosed with a temporo‐frontal network and 30% with fronto‐temporal network. The first group was defined as patients having the seizure onset zone in structures within the temporal lobe (hippocampus, amygdala, temporal pole, lateral temporal neocortex). In this group (67 patients), the most frequent sublobar frontal propagation was the orbitofrontal cortex (26) followed by cingulate cortex (9), orbitofrontal cortex and cingulate cortex (5), dorsolateral prefrontal (6), and mesial and dorsolateral frontal (15 patients), not determined in 9 patients (Table S1). The second group included patients suffering from frontal lobe epilepsy with a seizure onset zone situated in the orbitofrontal cortex, cingulate cortex, and dorsolateral prefrontal cortex. In the second group (33 patients), the most frequent temporal sub‐lobar propagation sites were the amygdala (12), the hippocampus (3), the mesio‐temporal (4), and all temporal (14). In 9 patients, there was no specific information related to the seizure onset zone (temporal or frontal); however, a fronto‐temporal network was described using only noninvasive data. An overview of the frontal and temporal propagation is available in Figure 4.

**FIGURE 5:**
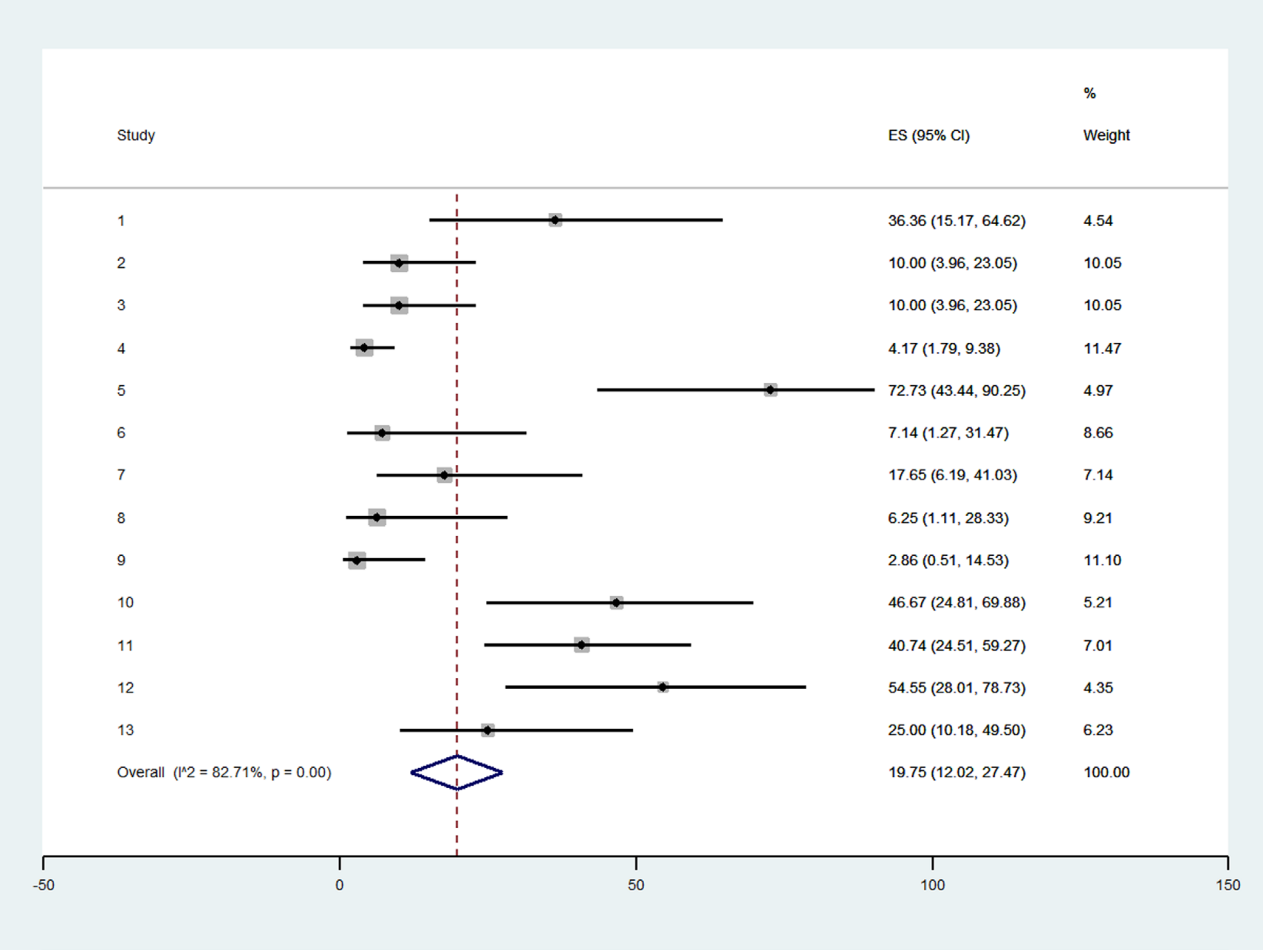
Meta‐analysis showing the prevalence of patients with fronto‐temporal network involvement in each study.

The meta‐analysis shows the total prevalence of fronto‐temporal network involvement was 19.75%, CI 12.02–27.47, and a high heterogeneity of 82.71% (Figure 5).

From the total of 109 patients, 24 (22%) did not receive any kind of surgery or the information related to surgical resection was not available. Eighty‐five patients (88%) underwent surgery. Forty‐two patients (49%) underwent temporal lobe resection, of which 7 (8%) underwent selective amygdalo‐hippocampectomy or lesionectomy (e.g., encephalocele resection) and 29 (34%) underwent resection in the frontal lobe. Fourteen patients (16%) underwent a fronto‐temporal resection (multilobar), most of them (12%–14%) include OFC and amygdala and/or temporal pole.

For 40 patients (47%), there was no information related to pathology (unspecific, not reported or patients were not operated on). Focal cortical dysplasia was found in 36 patients (42%), hippocampal sclerosis in 11 patients (13%), LEAT in 8 patients (9%), cavernoma in 3 patients (4%), temporal pole encephalocele in 2 patients (2%), glial scar in 4 patients (5%), hippocampal sclerosis and FCD, mMCD and neuronal heterotopia in 1(1%) patient each.

Fifty‐eight patients (68%) were seizure‐free (Engel IA) after surgery with a follow‐up of more than 12 months, 9 (10%) patients were classified as Engel I. There was no significant difference between seizure freedom in uni versus bilobar resections (*p* = .28) in none of the subgroups.

## 4 DISCUSSION

In this systematic review, we identified 40 articles from which we selected 109 patients with temporal (76 patients) and frontal (33 patients) lobe epilepsy and an epileptogenic network comprising the temporo‐frontal/frontal‐temporal connection. Patients were diagnosed with drug‐resistant epilepsy of structural etiology and underwent presurgical evaluation consisting of video‐EEG and/or stereo‐EEG seizure recordings and brain MRI that made possible reliable anatomo‐electro‐clinical correlations.

In our search, we found that the prevalence of the temporo‐frontal/frontal‐temporal network involvement in the 109 patients reported in the articles included was 19.75% (Figure 5). This finding is surprising given the fact that our search strategy focused on this particular network. However, this is explained by the fact that we included various types of articles such as those describing particular ictal signs (ictal swearing, ictal urination, and specific automatisms) which could be also associated with other ictal networks. For example, the 13th article (Table 1) Kuba et al., 2013 includes 120 patients and only 5 of those patients show RINCH automatisms associated with a fronto‐temporal network. To the best of our knowledge, there is no article specifically investigating the temporo‐frontal involvement in temporal lobe epilepsy, so the closest concept is temporal plus epilepsy. In this setting, the prevalence of temporal plus in temporal lobe epilepsy patients is reported around 10%, out of which 3.6% for suprasylvian involvement.^3^ This could explain the low prevalence that we found despite specific search.

In these cases, the ictal semiology was described in relation to the frontal/temporal ictal spread and we have identified that the automatisms are the most common features among these patients (73%) followed by elementary motor signs (47%) and hyperkinetic behavior (39%).

Among the automatisms (gestural, oroalimentary, vocal, or verbal), we identified specific ones associated with the frontal lobe spread. The rhythmic ictal non‐clonic hand motion (RINCH) is described as a sign of ictal spread from the temporal lobe in various regions of the contralateral frontal lobe, mainly the orbitofrontal cortex and anterior cingulate gyrus.^8^ Second, verbal automatisms particularly coprolalia occur in both temporal or orbitofrontal epilepsy and have a limited lateralizing value to the nondominant hemisphere but can be triggered by seizures from either hemisphere. It involves the activation of the paralimbic temporal–orbitofrontal network.^9^

Furthermore, the hyperkinetic behavior comprises an excessive amount and speed of motor movements with increased rate in motor sequences in line with the common terminology.^7^ This type of complex motor manifestations could be preceded by emotional (31%) and autonomic signs (38%). The underlying mechanism of hyperkinetic behavior was studied and revealed a change in connectivity/coupling between the dorsoanterior insular lobe, mesial frontal lobes (SMA/MCC), and the bilateral heads of the caudate nuclei.^10^ However, it has been also reported that the hyperkinetic behavior when following an intense emotional subjective manifestation (intense fear or anxiety) coupled also with facial expression of fear/horror is related to a disbalance in the limbic system, particularly the decoupling between the orbitofrontal cortex and the amygdala.^11^ Finally, we could explain the hyperkinetic behavior in temporal lobe seizures as a mark of the frontal ictal propagation as the frontal lobe, particularly the mesial‐prefrontal region could be a final common pathway responsible for this specific objective clinical sign. The anatomical basis sustaining the functional, electrophysiological ictal changes comprises three main white matter tracts. The uncinate fasciculus connects the temporo‐polar region with the frontal basal regions (e.g., BA47), the inferior frontal‐occipital fasciculus (IFOF) links the superior temporal gyrus to the ventral and lateral prefrontal cortex (BA 44), and the arcuate fasciculus links the caudal temporal with the posterior ventral and dorsal‐lateral prefrontal cortex.^12^

The frontal ictal propagation in temporal lobe epilepsy patients also has an impact in the surgical management. According to our review, almost 17% of the patients underwent frontal‐temporal resection but we did not find a significant association with postsurgical seizure freedom. This is different than recent studies showing that multilobar resections have higher changes to render seizure freedom in temporal plus epilepsy patients.^13^ We could explain this by the fact that in this review, we focused on seizure semiology and 25% of the included patients did not undergo surgery. Additionally, the group of patients receiving both fronto‐temporal resections is small and this could be a reason for not achieving the statistical significance. Finally, in the patients selected, the fronto‐temporal/temporo‐frontal network involvement does not necessarily imply an epileptogenic zone. This means that the temporal/frontal propagation could be part of the symptomatogenic zone not requiring resection for a good postsurgical outcome.

The cluster analysis shows a first cluster containing emotional, grimacing, autonomic, cognitive, and hyperkinetic signs with high association coefficients in all subgroups (Figure 3). This cluster of emotional‐autonomic‐hyperkinetic features is suggestive of the involvement of limbic structures, such as hippocampus, amygdala, orbitofrontal cortex within the frontal‐temporal network. The second cluster consisted of automatisms, such as gestural (RINCH), distal, mimicking (laughter/crying), oroalimentary, or verbal (ictal swearing) as the only symptom (Figure 3) or associated with motor elementary signs. These complex motor behaviors are frequently seen in the temporal lobe, temporal‐perisylvian, and temporo‐frontal seizures.^14^ Finally, the last cluster is that of elementary motor signs which are a mark of an extended network, affecting areas outside the limbic system of temporo‐frontal or fronto‐temporal seizures and are associated with large resections.

We found a statistically significant association between motor elementary signs and multilobar resection in the whole group and fronto‐temporal subgroup analysis in line with previous studies showing that motor elementary signs are most frequently present in temporal plus epilepsy.^15^ From a practical, clinical, point of view, this means that in patients with temporal/frontal epilepsy and motor elementary signs (tonic/clonic limb/facial movements), the SEEG exploration should take into account an extended network, fronto‐temporal or temporo‐frontal, and a multilobar resection could be necessary to maximize the postsurgical benefit. However, seizure semiology should be always interpreted integrated with other presurgical noninvasive information, particularly imaging and epilepsy etiology. There are particular etiologies such as post‐traumatic/post‐stroke lesions with low intrinsic epileptogenicity able to entail large, multilobar epileptogenic networks, particularly temporal or frontal.^16^, ^17^

There are several limitations to this review. Firstly, patient selection could introduce a bias since several articles included in this review investigated the cortical network of a specific clinical sign such as the automatisms that could generate an overrepresentation of this clinical manifestation. Secondly, there are only 58% of the studies included in which the confidence of the localization of the epileptogenic zone is very high (patients are seizure‐free Engel IA post‐surgery), which gives an overall moderate reliability according to GRADE system scoring. There is a great heterogeneity among data in each included study depending on its purpose. For example, the semiology was described in detail if the focus was on anatomo‐clinical correlation or it was brief if the objective was postsurgical seizure outcome. Finally, since not all patients underwent SEEG and in those that were invasively explored we quantified fronto‐temporal propagation not specifically fronto‐temporal epileptogenic zone, the lack of statistically significant results comparing uni vs. multilobar resection should be carefully interpreted.

## 5 CONCLUSIONS

The concept of temporo‐frontal/fronto‐temporal network in temporal or frontal epilepsy has a clinical relevance being associated with a specific cluster of signs and symptoms, such as emotional, autonomic, hyperkinetic behavior, grimacing, and cognitive. However, based on semiology, one cannot distinguish between fronto‐temporal and temporo‐frontal cases—at the individual patient levels. To answer the review's question, patients receiving multilobar resections (all group and fronto‐temporal subgroup) displayed statistically significant more frequent elementary motor signs. These findings imply that patients with drug‐resistant epilepsy and seizure semiology suggesting a fronto‐temporal network that has motor elementary signs should undergo invasive exploration with the probability of a tailored multilobar resection for reaching seizure freedom. However, these findings should in the future be confirmed by prospective studies.

## FUNDING INFORMATION

No funding.

## CONFLICT OF INTEREST STATEMENT

Authors have no conflict of interest to declare.

6

**Test yourself**

1. What are the main clinical features identified by the cluster analysis of seizures involving fronto‐temporal network?
  - Elementary motor, visual, olfactory, and cognitive
  - Emotional, hyperkinetic, autonomic, and grimacing
  - Hyperkinetic behavior, visual, emotional, and somatosensory
  - Autonomic, hyperkinetic, automatisms, and auditory
  - Complex somatosensory, visual, autonomic, and grimacing
2. What are the main brain regions included in frontal‐temporal network?
  - Temporal neocortex—orbitofrontal cortex—frontal pole
  - Temporal basal region—insula—prefrontal cortex
  - Temporal pole—frontal operculum—premotor cortex
  - Hippocampus—temporal operculum—premotor cortex
  - Medial temporal region—orbitofrontal cortex—cingulate cortex
3. What is the clinical sign most frequently predicting a multilobar resection?
  - Bilateral tonic—clonic contraction
  - Epileptic spasms
  - Autonomic signs
  - Elementary motor signs
  - Hyperkinetic behavior

*Answers may be found in the* supporting information.

## Supporting information


Appendix S1.

## References

[1] Lieb JP , Dasheiff RM , Engel J . Role of the frontal lobes in the propagation of mesial temporal lobe seizures. Epilepsia. 1990;32(6):822–837. 10.1111/J.1528-1157.1991.TB05539.X 1743154

[2] Blumenfeld H , Rivera M , McNally KA , Davis K , Spencer DD , Spencer SS . Ictal neocortical slowing in temporal lobe epilepsy. Neurology. 2004;63(6):1015–1021. 10.1212/01.WNL.0000141086.91077.CD/SUPPL_FILE/E3.DOC 15452292

[3] Barba C , Rheims S , Minotti L , Guénot M , Hoffmann D , Chabardès S , et al. Temporal plus epilepsy is a major determinant of temporal lobe surgery failures. Brain. 2016;139(2):444–451. 10.1093/BRAIN/AWV372 26700686

[4] Bartolomei F , Chauvel P , Wendling F . Epileptogenicity of brain structures in human temporal lobe epilepsy: a quantified study from intracerebral EEG. Brain. 2008;131(7):1818–1830. 10.1093/brain/awn111 18556663

[5] Kahane P , Barba C , Rheims S , Job‐Chapron AS , Minotti L , Ryvlin P . The concept of temporal ‘plus’ epilepsy. Rev Neurol (Paris). 2015;171(3):267–272. 10.1016/j.neurol.2015.01.562 25748333

[6] Gröppel G , Kapitany T , Baumgartner C . Cluster analysis of clinical seizure semiology of psychogenic nonepileptic seizures. Epilepsia. 2000;41(5):610–614. 10.1111/j.1528-1157.2000.tb00216.x 10802768

[7] Beniczky S , Tatum WO , Blumenfeld H , Stefan H , Mani J , Maillard L , et al. Seizure semiology: ILAE glossary of terms and their significance. Epileptic Disord. 2022;24(3):447–495. 10.1684/EPD.2022.1430 35770761

[8] Kuba R , Musilová K , Vojvodič N , Tyrlíková I , Rektor I , Brázdil M . Rhythmic ictal nonclonic hand (RINCH) motions in temporal lobe epilepsy: invasive EEG findings, incidence, and lateralizing value. Epilepsy Res. 2013;106(3):386–395. 10.1016/J.EPLEPSYRES.2013.06.015 23928193

[9] Massot‐Tarrús A , Mousavi SR , Dove C , Hayman‐Abello SS , Hayman‐Abello B , Derry PA , et al. Coprolalia as a manifestation of epileptic seizures. Epilepsy Behav. 2016;60:99–106. 10.1016/j.yebeh.2016.04.040 27195785

[10] Wang X , Hu WH , Zhang C , Shao XQ , Sang L , Zheng Z , et al. Neural networks underlying hyperkinetic seizures: a quantitative PET and SEEG study. Epilepsy Behav. 2021;122:108130. 10.1016/J.YEBEH.2021.108130 34153637

[11] Bartolomei F , Trébuchon A , Gavaret M , Régis J , Wendling F , Chauvel P . Acute alteration of emotional behaviour in epileptic seizures is related to transient desynchrony in emotion‐regulation networks. Clin Neurophysiol. 2005;116(10):2473–2479. 10.1016/J.CLINPH.2005.05.013 16125458

[12] Binney RJ , Parker GJM , Lambon Ralph MA . Convergent connectivity and graded specialization in the rostral human temporal lobe as revealed by diffusion‐weighted imaging probabilistic tractography. J Cogn Neurosci. 2012;24(10):1998–2014. 10.1162/JOCN_A_00263 22721379

[13] Barba C , Rheims S , Minotti L , Grisotto L , Chabardès S , Guenot M , et al. Surgical outcome of temporal plus epilepsy is improved by multilobar resection. Epilepsia. 2022;63(4):769–776. 10.1111/EPI.17185 35165888

[14] Wang Y , Wang X , Mo JJ , Sang L , Zhao BT , Zhang C , et al. Symptomatogenic zone and network of oroalimentary automatisms in mesial temporal lobe epilepsy. Epilepsia. 2019;60(6):1150–1159. 10.1111/EPI.15457 31095733

[15] Andrade‐Machado R , Benjumea‐Cuartas V . Temporal plus epilepsy: Anatomo‐electroclinical subtypes. Iran J Neurol. 2016;15(3):153. 27648177 PMC5027151

[16] Fierain A , McGonigal A , Lagarde S , Catenoix H , Valton L , Rheims S , et al. Stereoelectroencephalography (SEEG) and epilepsy surgery in posttraumatic epilepsy: a multicenter retrospective study. Epilepsy Behav. 2020;112:107378. 10.1016/J.YEBEH.2020.107378 32835959

[17] Marchi A , Pennaroli D , Lagarde S , McGonigal A , Bonini F , Carron R , et al. Epileptogenicity and surgical outcome in post stroke drug resistant epilepsy in children and adults. Epilepsy Res. 2019;155:106155. 10.1016/J.EPLEPSYRES.2019.106155 31252221

[18] Rheims S , Ryvlin P , Scherer C , Minotti L , Hoffmann D , Guenot M , et al. Analysis of clinical patterns and underlying epileptogenic zones of hypermotor seizures. Epilepsia. 2008;49(12):2030–2040. 10.1111/J.1528-1167.2008.01675.X 18503559

[19] Bartolomei F , Lagarde S , Lambert I , Trébuchon A , Villalon SM , McGonigal A , et al. Brain connectivity changes during ictal aggression (a strangulation attempt). Epileptic Disord. 2017;19(3):367–373. 10.1684/EPD.2017.0925 28830845

[20] Proserpio P , Cossu M , Francione S , Gozzo F , Lo Russo G , Mai R , et al. Epileptic motor behaviors during sleep: anatomo‐electro‐clinical features. Sleep Med. 2011;12(SUPPL 2):S33–S38. 10.1016/J.SLEEP.2011.10.018 22136897

[21] Nobili L , Francione S , Cardinale F , Lo Russo G . Epileptic nocturnal wanderings with a temporal lobe origin: a stereo‐electroen‐cephalographic study. SLEEP Neurol Disord. 2002;25(6):669–671. 12224845

[22] Biraben A , Taussig D , Thomas P , Even C , Vignal JP , Scarabin JM , et al. Fear as the main feature of epileptic seizures. J Neurol Neurosurg Psychiatry. 2001;70(2):186–191. 10.1136/jnnp.70.2.186 11160466 PMC1737203

[23] Bartolomei F , Cosandier‐Rimele D , McGonigal A , Aubert S , Régis J , Gavaret M , et al. From mesial temporal lobe to temporoperisylvian seizures: a quantified study of temporal lobe seizure networks. Epilepsia. 2010;51(10):2147–2158. 10.1111/J.1528-1167.2010.02690.X 20738379

[24] Vaugier L , McGonigal A , Lagarde S , Trébuchon A , Szurhaj W , Derambure P , et al. Hyperkinetic motor seizures: a common semiology generated by two different cortical seizure origins. Epileptic Disord. 2017;19(3):362–366. 10.1684/EPD.2017.0932 28830844

[25] Vaugier L , Aubert S , McGonigal A , Trébuchon A , Guye M , Gavaret M , et al. Neural networks underlying hyperkinetic seizures of ‘temporal lobe’ origin. Epilepsy Res. 2009;86(2–3):200–208. 10.1016/J.EPLEPSYRES.2009.06.007 19619985

[26] Bartolomei F , Wendling F , Vignal JP , Chauvel P , Liégeois‐Chauvel C . Neural networks underlying epileptic humming. Epilepsia. 2002;43(9):1001–1012. 10.1046/J.1528-1157.2002.48501.X 12199725

[27] Bottan JS , Suller Marti A , Parrent AG , MacDougall KW , McLachlan RS , Burneo JG , et al. Seizure freedom in temporal plus epilepsy surgery following stereo‐electroencephalography. Can J Neurol Sci. 2020;47(3):374–381. 10.1017/CJN.2020.26 32036799

[28] Nobili L , Cossu M , Mai R , Tassi L , Cardinale F , Castana L , et al. Sleep‐related hyperkinetic seizures of temporal lobe origin. Neurology. 2004;62(3):482–485. 10.1212/01.WNL.0000106945.68292.DC 14872038

[29] Fong MWK , Sala‐Padro J , Bartley M , Dexter MAJ , Bleasel AF , Wong CH . The varied semiology of seizures in the context of small anterior temporal encephaloceles. Epileptic Disord. 2019;21(4):347–352. 10.1684/EPD.2019.1081 31366450

[30] Tassinari CA , Tassi L , Calandra‐Buonaura G , Stanzani‐Maserati M , Fini N , Pizza F , et al. Biting behavior, aggression, and seizures. Epilepsia. 2005;46(5):654–663. 10.1111/J.1528-1167.2005.58404.X 15857430

[31] Hogan RE , Rao VK . Hemifacial motor and crying seizures of temporal lobe onset: case report and review of electro‐clinical localisation. J Neurol Neurosurg Psychiatry. 2006;77(1):107–110. 10.1136/JNNP.2005.062554 16361607 PMC2117386

[32] Wang X , Hu W , Zhang K , Shao X , Ma Y , Sang L , et al. The Anatomo‐electrical network underlying Hypermotor seizures. Front Neurol. 2018;9:243. 10.3389/FNEUR.2018.00243 29695997 PMC5904199

[33] Xu C , Ma K , Zhang X , Yu T , Zhang G , Wang Y , et al. Ictal coprolalia occurs due to the activation of the temporal‐orbitofrontal network in patients with epilepsy. J Neurol Sci. 2020;409:116634. 10.1016/J.JNS.2019.116634 31864073

[34] Wong CH , Mohamed A , Larcos G , McCredie R , Somerville E , Bleasel A . Brain activation patterns of versive, hypermotor, and bilateral asymmetric tonic seizures. Epilepsia. 2010;51(10):2131–2139. 10.1111/J.1528-1167.2010.02723.X 21069905

[35] Tran TPY , Truong VT , Wilk M , Tayah T , Bouthillier A , Mohamed I , et al. Different localizations underlying cortical gelastic epilepsy: case series and review of literature. Epilepsy Behav. 2014;35:34–41. 10.1016/J.YEBEH.2014.03.024 24798408

[36] Loução de Amorim I , Pereira C , Sequeira J , Rocha H , Peralta AR , Rego R , et al. Gelastic seizures: a retrospective study in five tertiary hospital centres. Epileptic Disord. 2020;22(2):165–175. 10.1684/EPD.2020.1153 32364505

[37] Dede HÖ , Bebek N , Gürses C , Baysal‐Kıraç L , Baykan B , Gökyiğit A . Genital automatisms: reappraisal of a remarkable but ignored symptom of focal seizures. Epilepsy Behav. 2018;80:84–89. 10.1016/J.YEBEH.2017.12.023 29414563

[38] Agashe S , Lundstrom BN , Brinkmann BH , So E , Cascino GD , Gregg N , et al. Temporal encephalocele: an epileptogenic focus confirmed by direct intracranial electroencephalography. Epilepsy Behav Rep. 2023;22:100601. 10.1016/J.EBR.2023.100601 37122846 PMC10131120

[39] Liu Y , Wang S , Hong B , Wang H , Lin J , Shi J , et al. Electroclinical features of lateral and medial orbitofrontal epilepsy: a case series. Epileptic Disord. 2020;22(6):759–767. 10.1684/EPD.2020.1230 33337332

[40] Zhao B , Zhang C , Wang X , Wang Y , Mo J , Zheng Z , et al. Orbitofrontal epilepsy: distinct neuronal networks underlying electroclinical subtypes and surgical outcomes. J Neurosurg. 2020;135(1):255–265. 10.3171/2020.5.JNS20477 32823264

[41] Serletis D , Bulacio J , Alexopoulos A , Najm I , Bingaman W , González‐Martínez J . Tailored unilobar and multilobar resections for orbitofrontal‐plus epilepsy. Neurosurgery. 2014;75(4):388–397. 10.1227/NEU.0000000000000481 24991708

[42] Hedaya A , Ver Hoef L . ‘Amity seizures’: a previously unreported semiology localizing to a circuit between the right hippocampus and orbitofrontal area. Epilepsy Behav Rep. 2024;25:100649. 10.1016/J.EBR.2024.100649 38323089 PMC10844940

[43] Ishizaki T , Maesawa S , Nakatsubo D , Yamamoto H , Shibata M , Kato S , et al. Anatomo‐electro‐clinical correlations of hypermotor seizures with amygdala enlargement: hippocampal seizure origin identified using stereoelectroencephalography. Epilepsy Behav Case Rep. 2019;11:10–13. 10.1016/J.EBCR.2018.09.011 30591881 PMC6305660

[44] Sevy A , Gavaret M , Trebuchon A , Vaugier L , Wendling F , Carron R , et al. Beyond the lesion: the epileptogenic networks around cavernous angiomas. Epilepsy Res. 2014;108(4):701–708. 10.1016/J.EPLEPSYRES.2014.02.018 24661427

[45] Alsemari A , Alotaibi F , Baz S . Ictal kissing with subdural EEG recording. Epilepsy Behav Case Rep. 2013;1(1):79–84. 10.1016/J.EBCR.2013.05.001 25667835 PMC4150642

[46] Zhang H , Wang D , Ren L , Fan X , Shan Y , Zhao G . Ictal swearing network confirmed by stereoencephalography: a case report. Acta Neurochir. 2019;161(12):2499–2503. 10.1007/S00701-019-04091-0/METRICS 31707457

[47] Loddenkemper T , Foldvary N , Raja S , Neme S , Lüders HO . Ictal urinary urge: further evidence for lateralization to the nondominant hemisphere. Epilepsia. 2003;44(1):124–126. 10.1046/J.1528-1157.2003.26202.X 12581239

[48] Chibane IS , Boucher O , Dubeau F , Tran TPY , Mohamed I , McLachlan R , et al. Orbitofrontal epilepsy: case series and review of literature. Epilepsy Behav. 2017;76:32–38. 10.1016/J.YEBEH.2017.08.038 28928072

[49] Gurgenashvili K , Massey SL , Grant M , Piatt J , Legido A , Valencia I . Intracranial localisation of ictal urinary urge epileptogenic zone to the non‐dominant temporal lobe. Epileptic Disord. 2011;13(4):430–434. 10.1684/EPD.2011.0476 22258049

[50] Shihabuddin B , Abou‐Khalil B , Delbeke D , Fakhoury T . Orbito‐frontal epilepsy masquerading as temporal lobe epilepsy‐a case report. Seizure. 2001;10(2):134–138. 10.1053/SEIZ.2000.0477 11407958

[51] Roper SN , Gilmore RL . Orbitofrontal resections for intractable partial seizures. J Epilepsy. 1995;8(2):146–152. 10.1016/0896-6974(95)00023-7

[52] Tezer FI , Agan K , Borggraefe I , Noachtar S . Seizure semiology reflects spread from frontal to temporal lobe: evolution of hyperkinetic to automotor seizures as documented by invasive EEG video recordings. Epileptic Disord. 2013;15(3):338–341. 10.1684/EPD.2013.0597 23968850

[53] Yu T , Zhang G , Wang Y , Cai L , Zhou X , du W , et al. Surgical treatment of hypermotor seizures originating from the temporal lobe. Seizure. 2013;22(10):862–866. 10.1016/J.SEIZURE.2013.07.009 23927871

