# Supplementary material for: Ictal semiology in temporo‐frontal epilepsy: A systematic review and meta‐analysis
Source: Epileptic Disord. 2024 Dec 26;27(2):171–86. doi: 10.1002/epd2.20328 (PMC12067355; doi:10.1002/epd2.20328)
Supplement: Supplementary file 1 — Appendix S1. [file EPD2-27-171-s001.docx]

**TEST YOURSELF**

**Answers:**

1. **B**

2. **E**

3. **D**
